# Supplementary figures and images for: Long-Term Personalization of an In-Home Socially Assistive Robot for Children With Autism Spectrum Disorders
Source: Front Robot AI. 2019 Nov 6;6:110. doi: 10.3389/frobt.2019.00110 (PMC7805891; doi:10.3389/frobt.2019.00110)

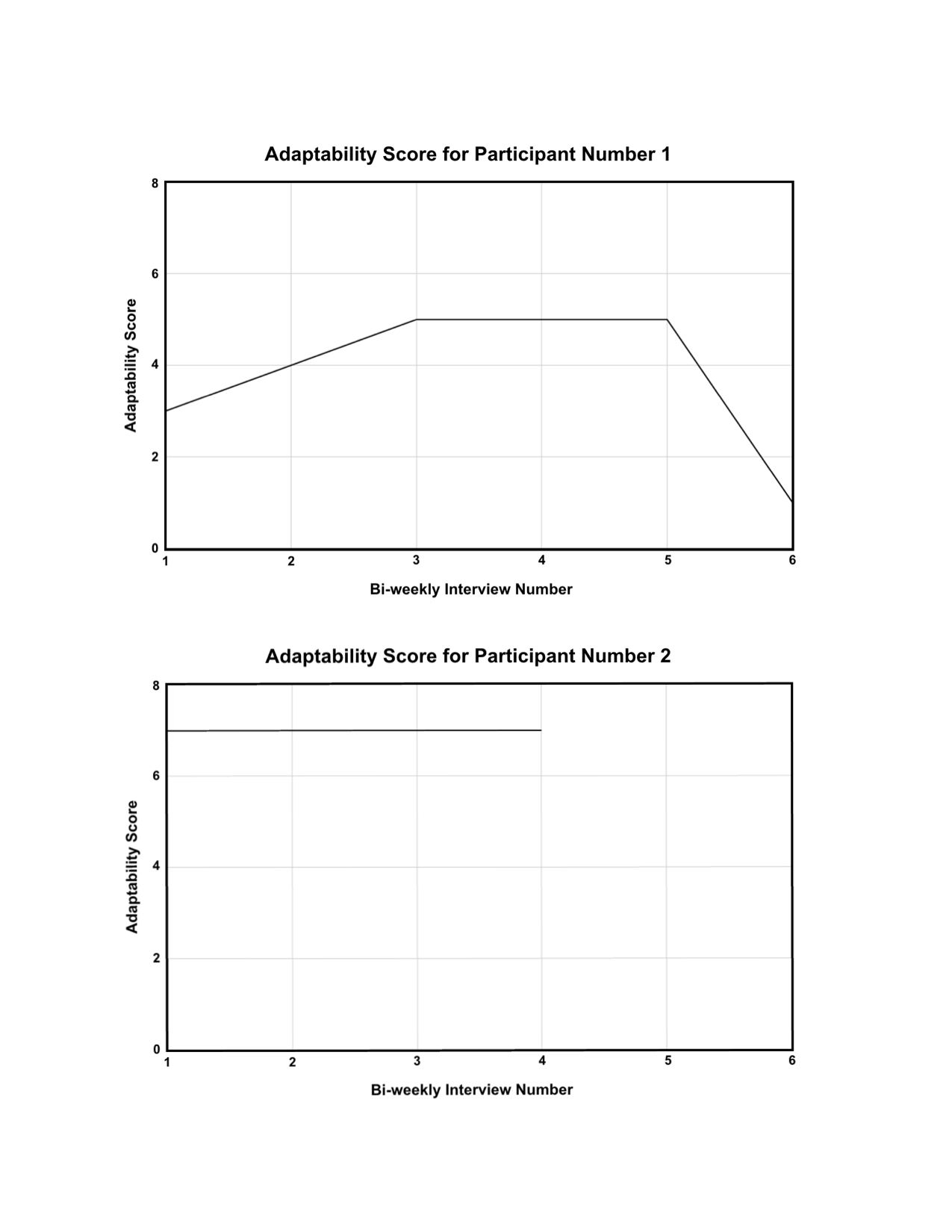

Supplement: Supplementary file 1 [file Image_1.PNG]

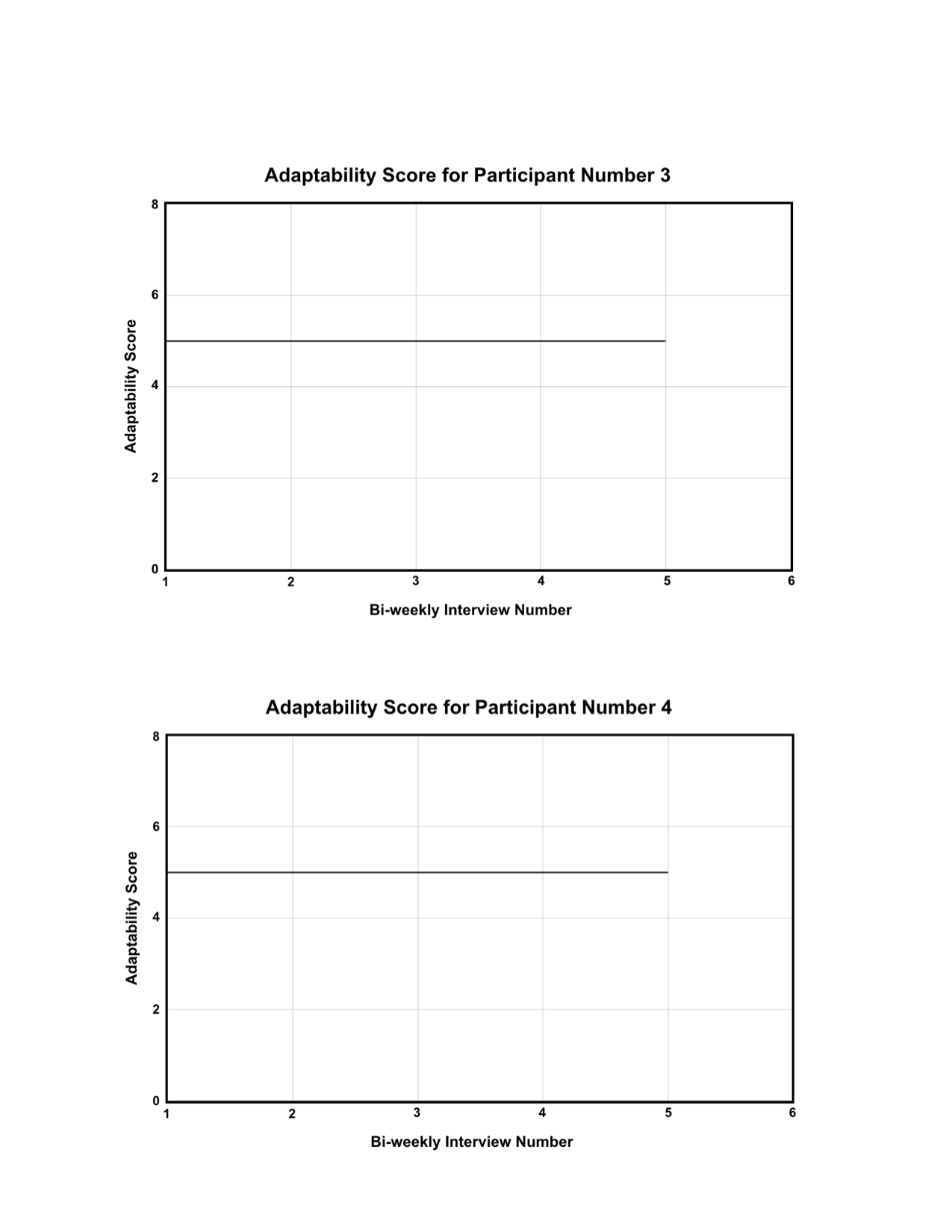

Supplement: Supplementary file 2 [file Image_2.PNG]

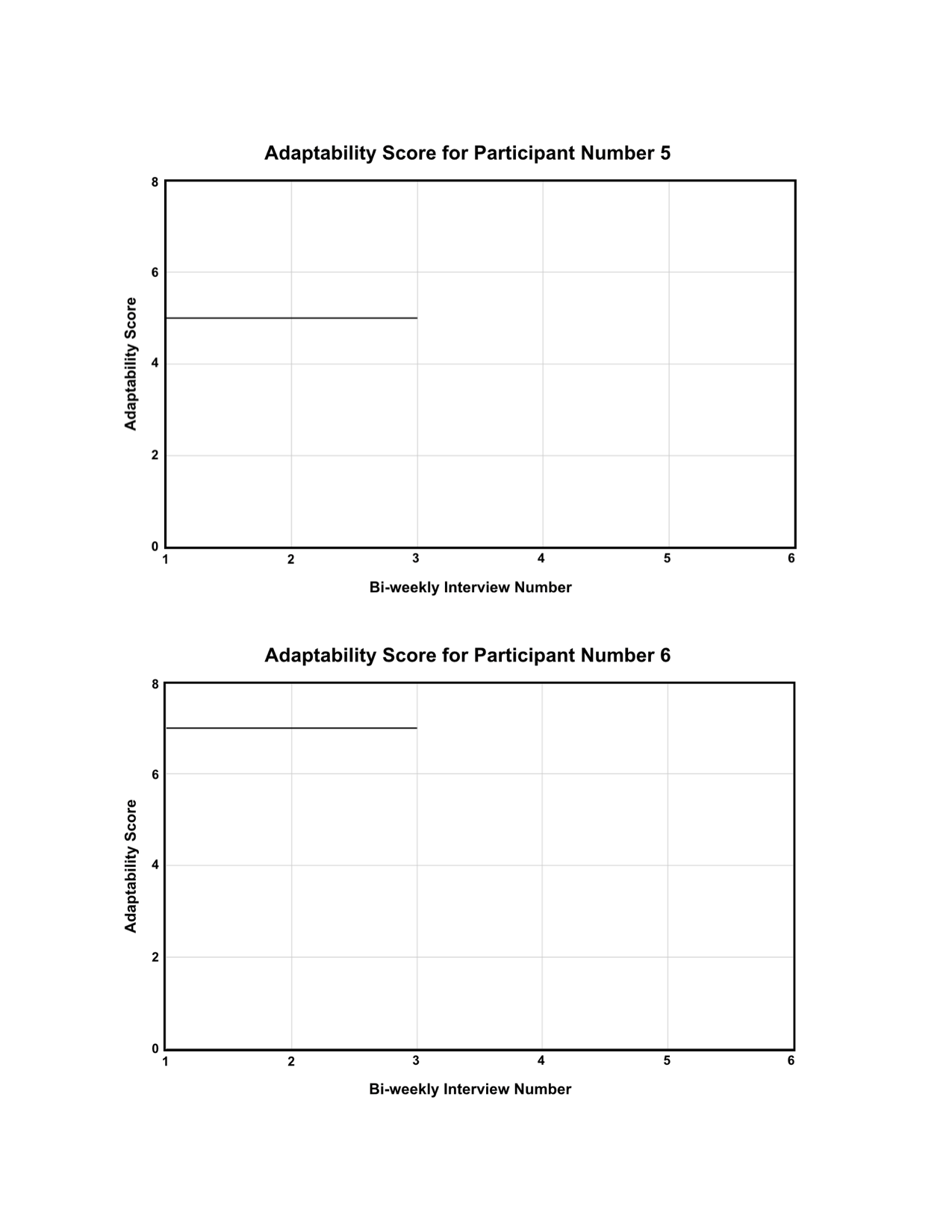

Supplement: Supplementary file 3 [file Image_3.PNG]

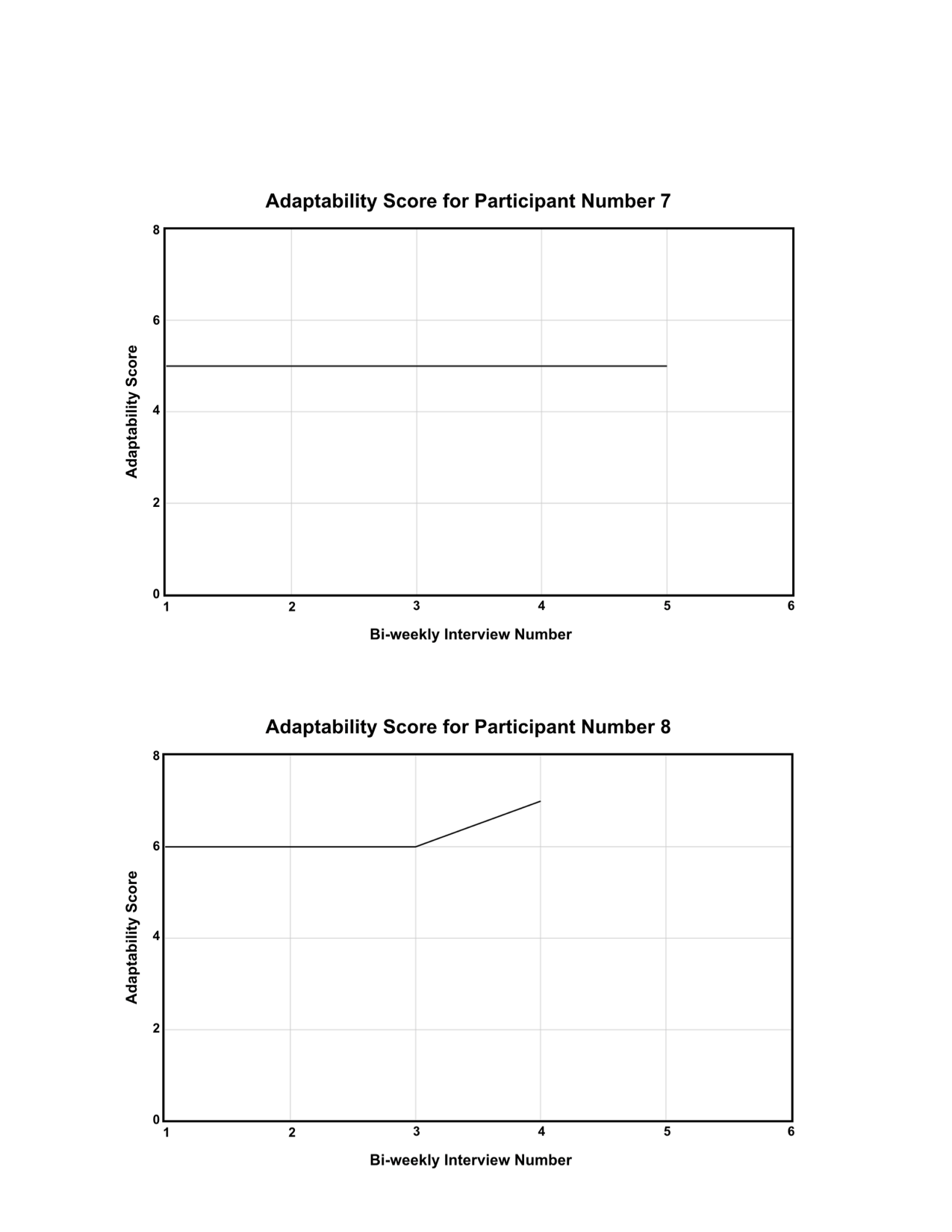

Supplement: Supplementary file 4 [file Image_4.PNG]

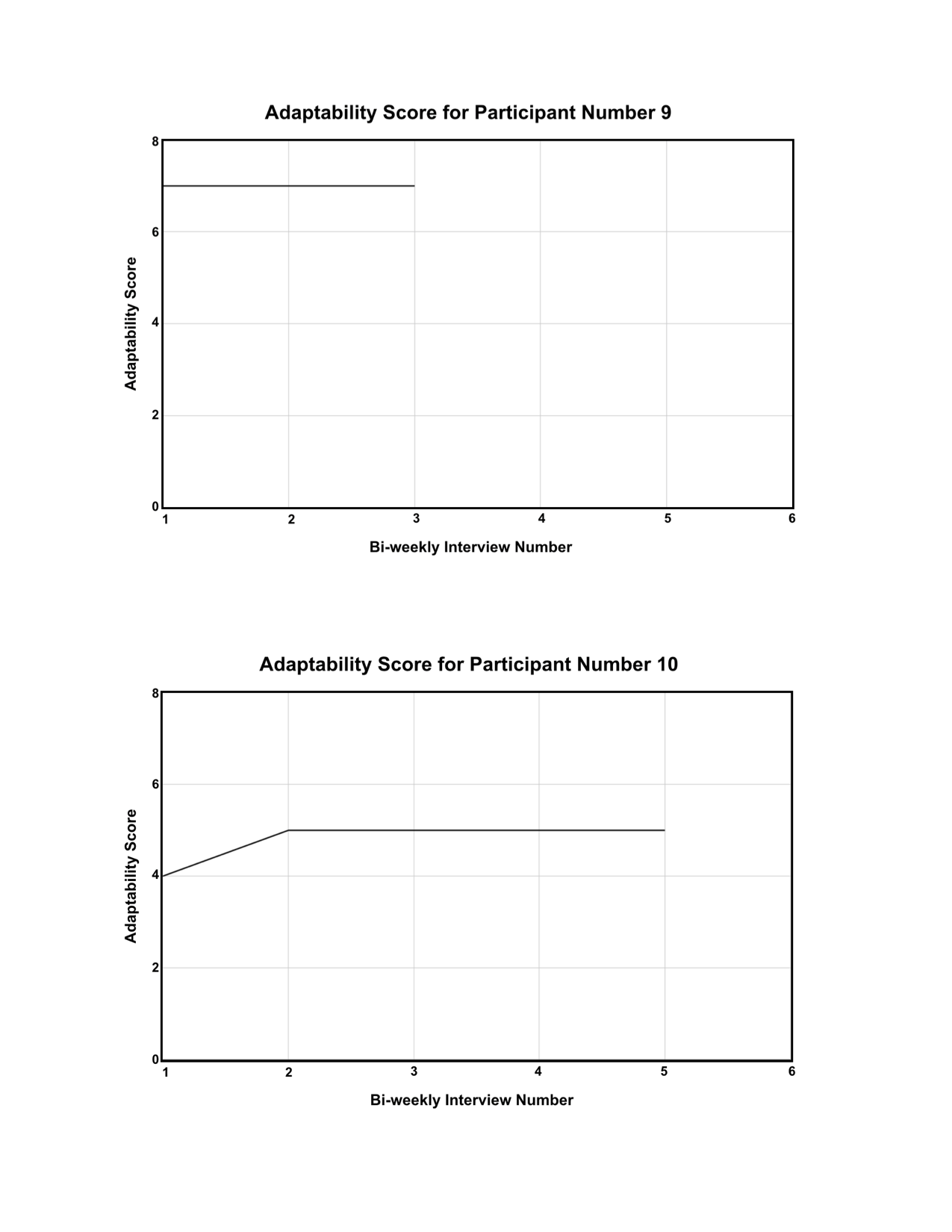

Supplement: Supplementary file 5 [file Image_5.PNG]

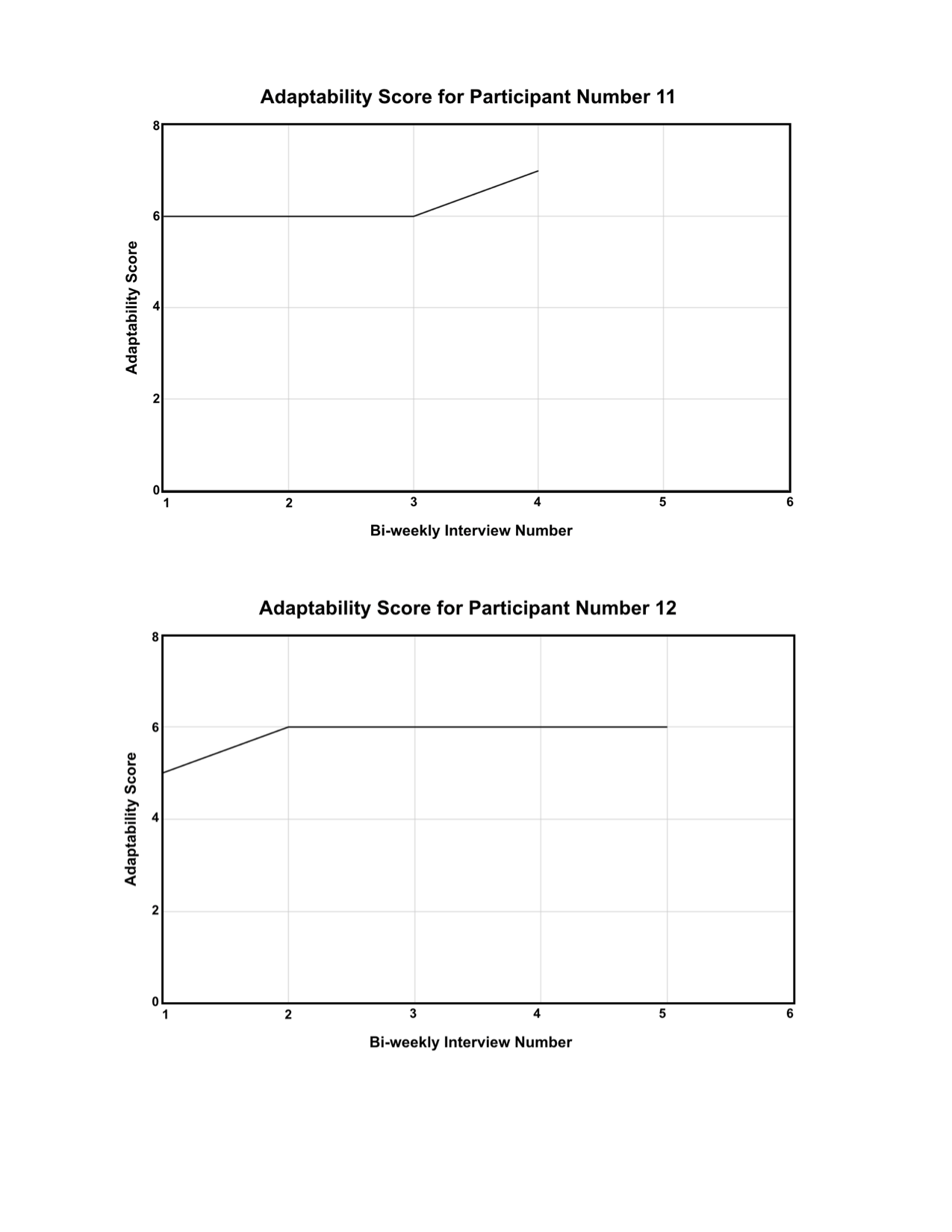

Supplement: Supplementary file 6 [file Image_6.PNG]

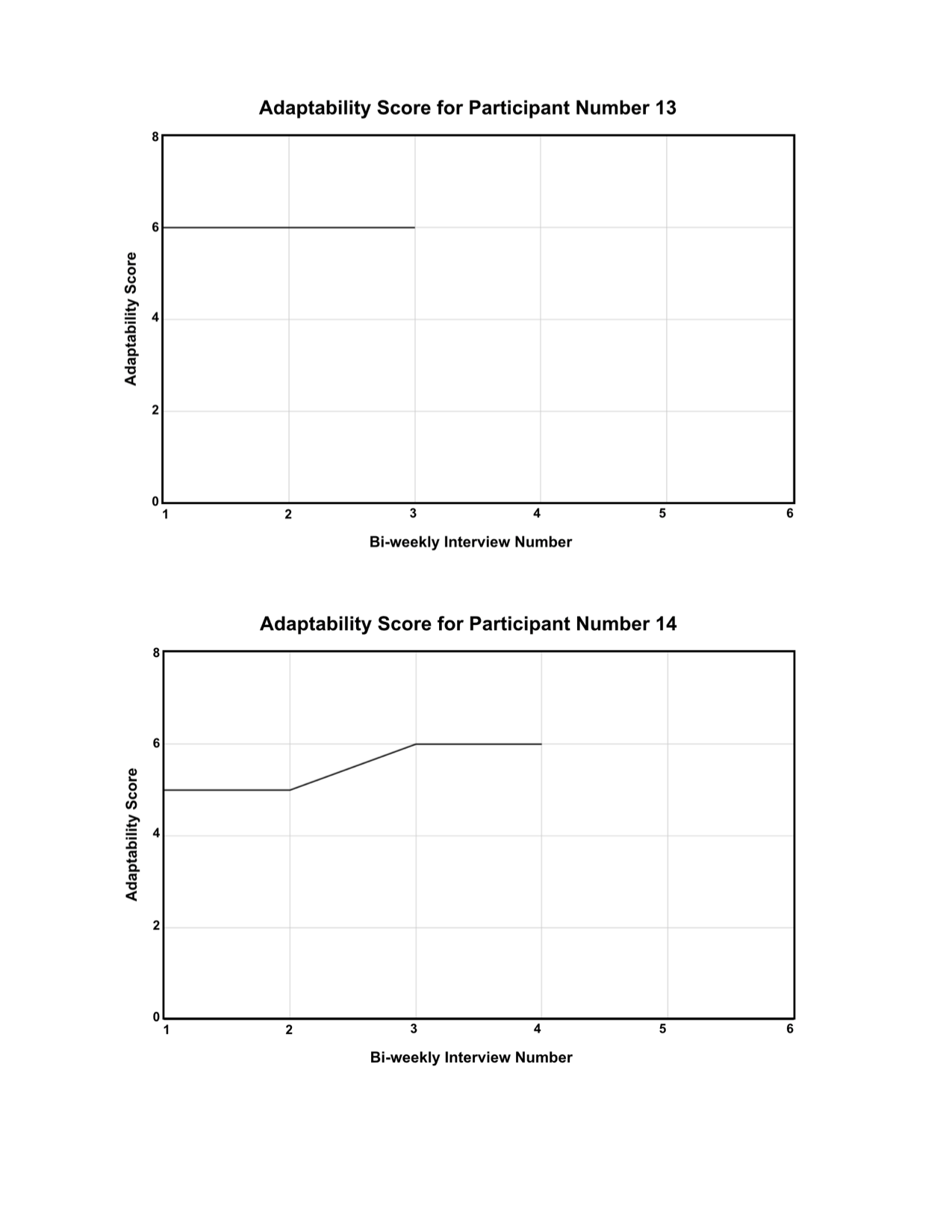

Supplement: Supplementary file 7 [file Image_7.PNG]

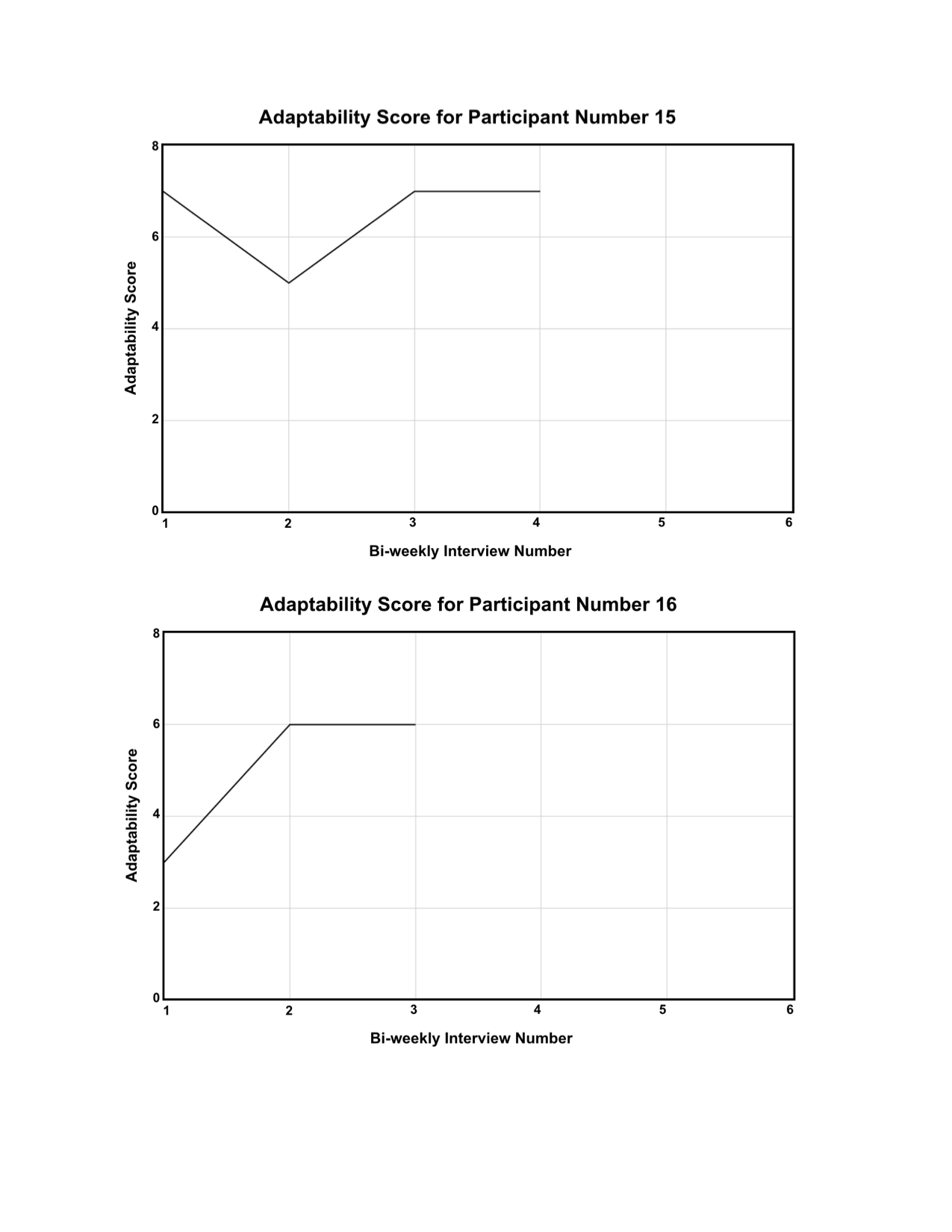

Supplement: Supplementary file 8 [file Image_8.PNG]

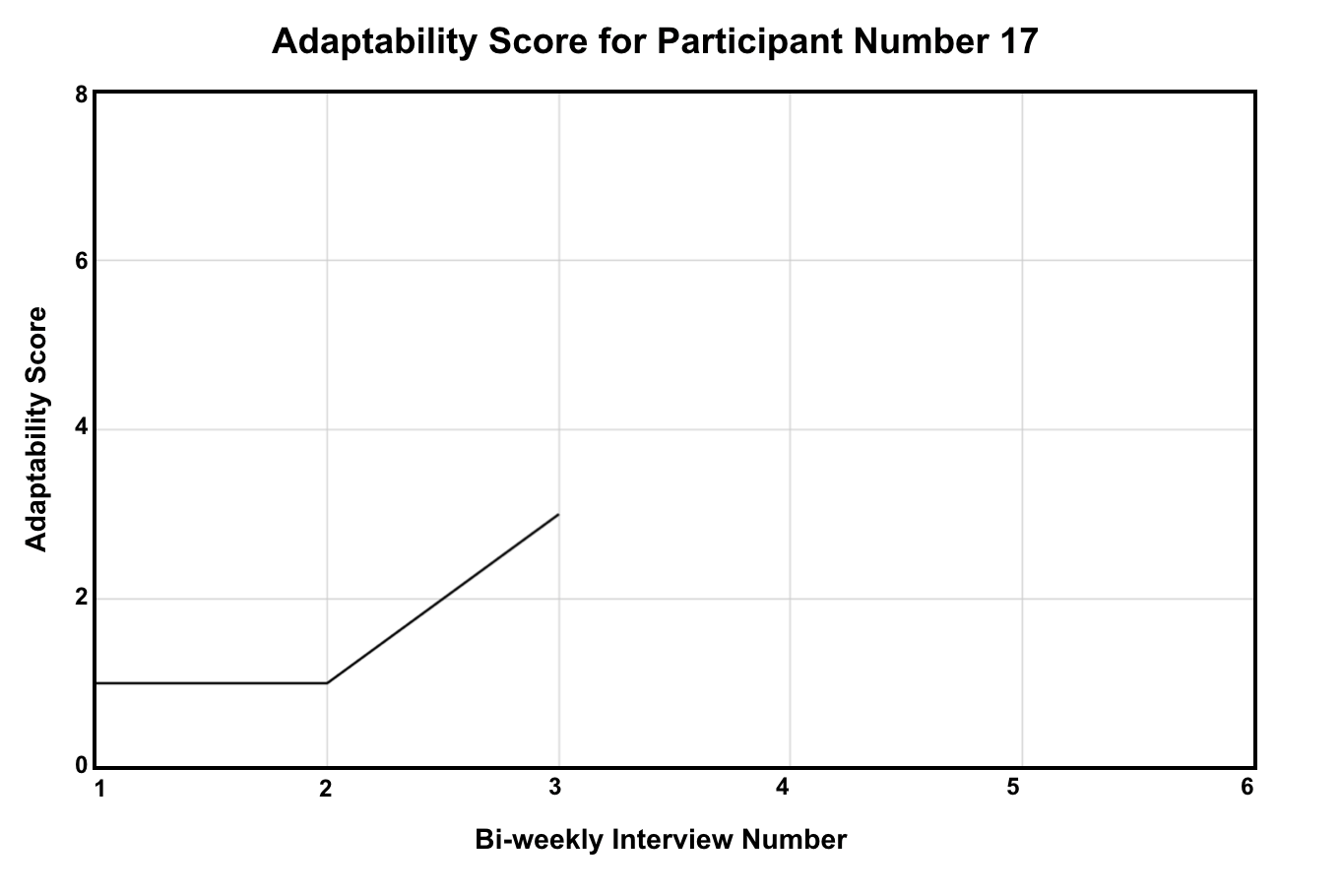

Supplement: Supplementary file 9 [file Image_9.PNG]

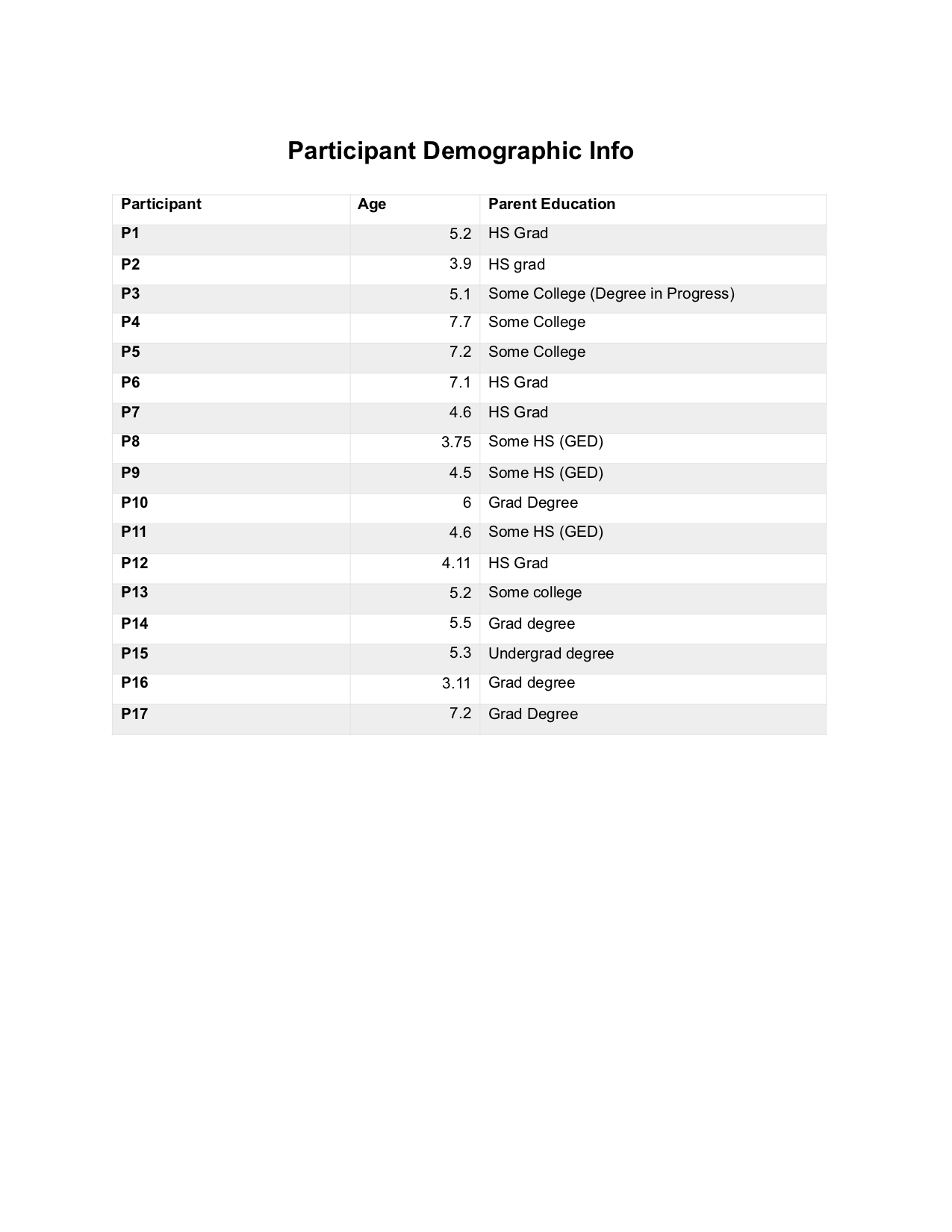

Supplement: Supplementary file 10 [file Image_10.PNG]
